# Temporal trends and forecasted mortality involving lung cancer with co-listed hypertension in U.S. adults, 2000–2035

**DOI:** 10.1186/s40959-026-00524-9

**Published:** 2026-06-20

**Authors:** Syeda Samia Fatima, Asma Naz, Muneeb Fareed, Muhammad Hussain, FNU Arsheen, Nisha Khalid, Muhammad Hassan, FNU Pirih, S M Aleem Hussain, Saifullah Khan, Hasibullah Aminpoor

**Affiliations:** 1 Department of Medicine, Jinnah Sindh Medical University, Karachi, Pakistan; 2 Department of Dow Medical College, Dow University of Health Sciences, Karachi, Pakistan; 3 People’s University of Medical and Health Sciences, Shaheed Bennazirabad, Pakistan; 4 Faculty of Medicine, Kabul University of Medical Sciences “Abu Ali Ibn Sina”, Kabul, Afghanistan

**Keywords:** Lung Cancer, Hypertension, Mortality, Disparities, Trends

## Abstract

**Background:**

Lung Cancer (LC) and Hypertension (HTN) frequently co-exist as a significant comorbidity with shared risk factors and pathophysiological mechanisms. We examined national trends and forecast future mortality involving LC co-listed with HTN among US adults from 2000 to 2025, with projections to 2035.

**Methods:**

Using CDC WONDER MULTIPLE Cause-of-Death, we conducted a retrospective analysis of LC and co-listed HTN mortality from 2000 to 2025 among adults aged ≥ 25 years. Age-adjusted mortality rates (AAMRs) were calculated, and joinpoint regression was utilized to estimate annual average percentage changes (AAPCs) with 95% confidence intervals (CIs). Auto-ARIMA and Prophet time-series models in R (v4.5.0) projected AAMRs through 2035, evaluated by root mean squared error (RMSE).

**Results:**

There were 305,878 reported deaths due to LC and co-listed HTN. The AAMR rose from 3.55 to 6.00 (AAPC 2.038; *p* < 0.0001). Men had higher mean AAMRs than women (mean AAMR: men 6.16; women 4.07). Among races, Non-Hispanic (NH) Black adults had the greatest mortality (mean AAMR: 7.38). Geographically, the South had the greatest burden (mean AAMR: 5.67) and non-metropolitan regions had higher mean AAMR than metropolitan regions (5.73 vs. 4.5). Most deaths (42.16%) occurred in decedents’ homes. The overall AAMR was projected to remain persistently elevated through 2035, particularly among men, NH White individuals, and in the Southern region with substantial uncertainty.

**Conclusion:**

Mortality associated with LC and co-listed HTN is an increasing healthcare burden in the US with marked demographic and regional disparities. Future projections indicate a persistent mortality burden, underscoring the need for targeted prevention strategies.

**Supplementary Information:**

The online version contains supplementary material available at https://doi.org/10.1186/s40959-026-00524-9.

## Introduction

Lung cancer (LC) remains the leading cause of cancer death in the United States (U.S), with an age-adjusted death rate of approximately 31.5 per 100,000 and an estimated 635,547 people living with lung and bronchus cancer in 2022 [1]. Global and country-level models project that LC incidence will increase in many regions by 2035, largely driven by population aging and demographic changes, alongside established risk factors such as tobacco use and air pollution [2, 3]. The macroeconomic toll of thoracic (tracheal, bronchus, lung) cancers is large; modeling estimates place LC among the top contributors to the global economic cost of cancer through 2050 [4]. Public research support for lung-cancer–focused work is substantial but variable; National Cancer Institute (NCI) reporting shows dedicated lung-cancer research line items within the NCI portfolio in recent budget factbooks [5]. Hypertension (HTN) is similarly ubiquitous: U.S. surveillance reports tens of thousands of deaths directly coded to hypertensive disease annually, and large swathes of the adult population are affected [6]. HTN’s direct U.S. healthcare costs have been estimated at tens of billions per year (historically ≈ U.S.$71 billion) and contribute markedly to overall cardiovascular expenditures [7]. Forecasts for cardiovascular risk-factor–driven costs (including HTN) project large increases in healthcare and productivity losses by mid-century [8].

HTN and LC intersect clinically and epidemiologically. Emerging reviews and mechanistic syntheses describe shared biological pathways—chronic inflammation, oxidative stress, endothelial dysfunction—and overlapping environmental exposures that plausibly link the two conditions [9]. Large population studies report high comorbidity; for example, national surveillance in China found a prevalence of HTN exceeding 50% among LC patients and observed an independent association between HTN and LC after multivariable adjustment [10]. Shared risk factors include smoking, air pollution, obesity, and age; their coexistence portends greater clinical complexity and higher mortality.

To our knowledge, national U.S. data describing long-term multiple-cause mortality trends involving lung cancer with co-listed hypertension remain limited. This retrospective analysis leverages nationally representative CDC surveillance data to address an important gap in the literature by describing mortality trends of LC–HTN comorbidity across longitudinal U.S. data. By providing quantification of the joint prevalence, temporal trends, and mortality patterns, the study offers a broader epidemiological perspective than previous studies, which investigated LC and co-listed HTN at a specific time point, in a regional population, or in a population outside the U.S. In doing so, it strengthens the evidence base for estimating current burden and informing projections of future disease impact, while also highlighting the need for integrated prevention strategies and cardio-oncology–focused care. Considering the above, the aim of the current study is to describe the epidemiology, temporal trends, and mortality associated with LC and co-listed HTN, using data from CDC.

## Methods

### Study population and setting

We conducted a retrospective analysis of national mortality data to evaluate temporal trends mortality involving lung cancer with co-listed hypertension using the Centers for Disease Control and Prevention Wide-Ranging Online Data for Epidemiologic Research (CDC WONDER) Multiple Cause-of-Death (MCD) database from 2000 to 2025. Deaths were identified from the CDC WONDER Multiple Cause-of-Death database when lung cancer (ICD-10 code C34) and hypertension (ICD-10 codes I10–I15) were co-listed on the death certificate within the multiple-cause-of-death fields, consistent with established epidemiological methodologies [11, 12]. This study adhered to the STROBE guidelines [13]. Institutional Review Board approval was not required due to the use of publicly available, de-identified data.

### Data extraction

Extracted variables included overall mortality, sex, race/ethnicity (Hispanic/Latino and non-Hispanic (NH) White, Black, Asian/Pacific Islander, geographic region, state, and urbanization status (urban ≥ 50,000 vs. rural < 50,000), as defined by the 2013 NCHS Urban–Rural Classification Scheme [14]. Place of death was classified as home, hospice, nursing home/long-term care facility, medical facility, or other/unknown.

### Statistical analysis

Age-adjusted mortality rates (AAMRs) per 100,000 population, standardized to the 2000 U.S. standard population, were calculated to assess temporal trends in LC with co-listed HTN mortality [15]. Mean AAMRs over the study period were calculated using the AVERAGE function in Microsoft Excel.

Joinpoint regression analysis was performed using log-transformed rates and standard weighted least squares settings [16]. A maximum of four joinpoints were permitted, and the optimal model was selected using the Monte Carlo permutation method implemented in the Joinpoint Regression Program. Annual percent change (APC) was estimated for each joinpoint-defined segment, while average annual percent change (AAPC) was calculated across the full study period (2000–2025) with corresponding 95% confidence intervals (CIs) were calculated for each segment, with statistical significance defined as a two-sided p-value < 0.05.

Mortality data were obtained from CDC WONDER. In accordance with CDC data suppression policies, death counts fewer than 10 were automatically suppressed to ensure confidentiality, and these suppressed values were handled automatically within the database during data extraction.

### Time-series forecasting

Time-series forecasting was conducted using Auto-ARIMA and Prophet models. The Auto-ARIMA model, implemented via the forecast package in R, automatically selected optimal parameters (p, d, q) based on the Akaike Information Criterion (AIC), with appropriate diagnostic and seasonal adjustments applied [17]. The Prophet model, developed by Meta Platforms, was implemented using the prophet package and decomposes time series into trend, seasonal, and irregular components using a generalized additive framework with automatically detected changepoints.

Data from 2000 to 2020 were used for model training, and 2021–2025 for validation. Model performance was assessed using a rolling one-step-ahead forecasting approach, with accuracy evaluated by root mean squared error (RMSE). The model with the lowest RMSE was selected and applied to the full dataset (2000–2025) to project age-adjusted mortality rates through 2035, with 95% confidence intervals.

Forecasts were interpreted cautiously and contextualized using previously established historical patterns rather than causal inference. All projections were generated under the assumption that historical trends would persist and may not account for future structural changes, policy interventions, healthcare advancements, or unforeseen external factors. Accordingly, language implying causality or deterministic prediction was avoided throughout the manuscript. All analyses were conducted using reproducible workflows implemented in R.

## Results

Between 2000 and 2025, mortality related to LC and co-listed HTN accounted for a total of 305,878 deaths among adults aged 25 years and older in the U.S. These deaths occurred across various locations in the country, with 28.49% taking place in medical facilities, 42.16% at the decedents’ homes, 17.03% in nursing homes, 7.47% in hospice facilities, and 4.68% at other locations (Supplemental Table 2).

### Annual trends

**Fig. 1:**
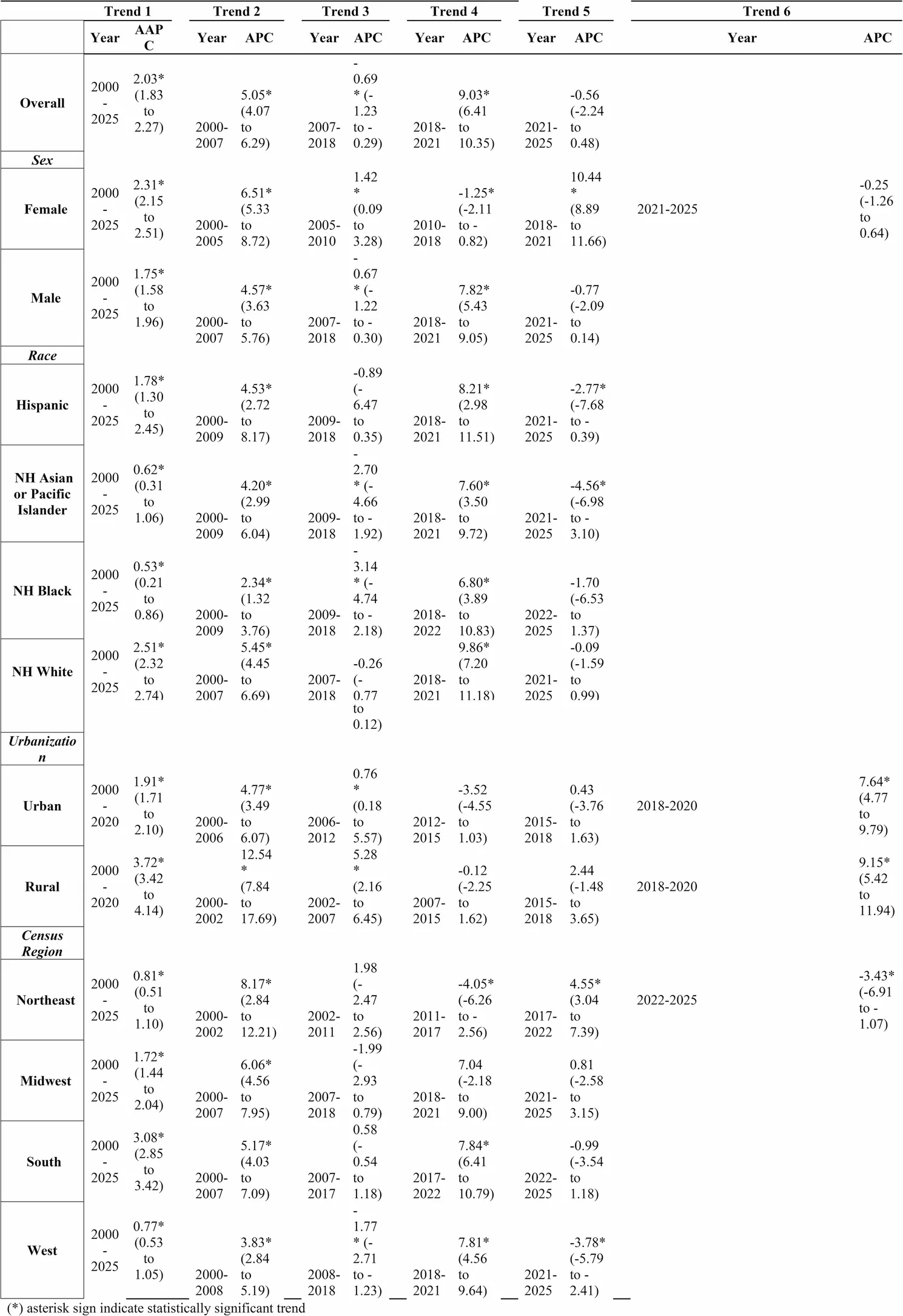
Table 1. AAMR and AAPC for lung cancer with co-listed hypertension among U.S. adults aged ≥ 25 years, 2000 to 2025

**Fig. 2:**
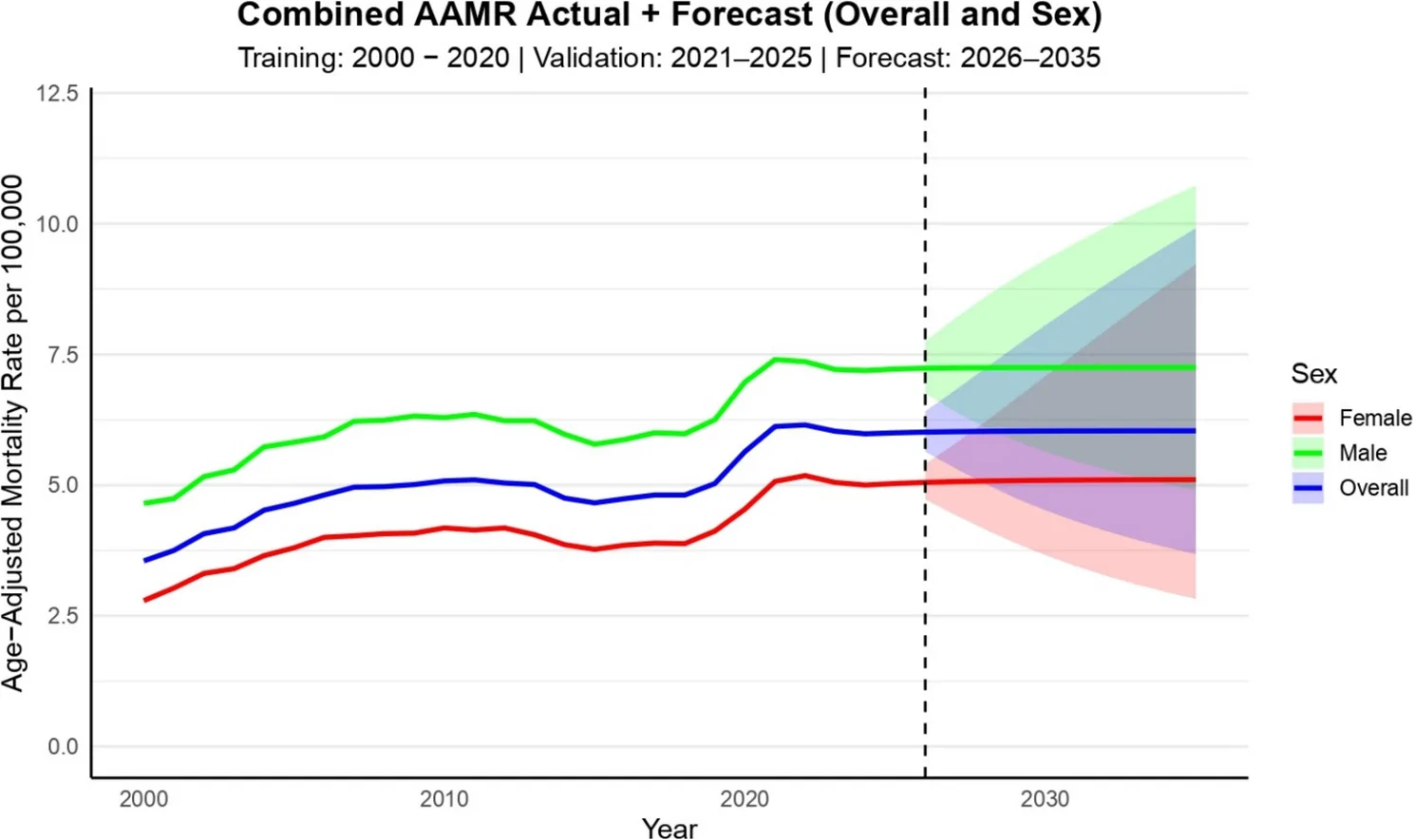
Lung cancer and co-listed hypertension AAMR stratified by overall and sex per 100,000 population

The overall AAMR for lung cancer with co-listed hypertension increased from 3.55 in 2000 to 6.00 in 2025, with a significant overall increase observed across the study period (AAPC:2.038 95% CI: 1.83 to 2.27; p value < 0.001) (Fig. 1 and 2) (Supplemental Table 1).

By 2035, the overall AAMR is projected to reach 6.03 (95% CI: 3.67 to 9.91) (Supplemental Table 9).

### Stratified by sex

The AAMR for both men and women increased from 2000 to 2025. Among men, the AAMR increased significantly from 4.65 (95% CI: 4.49 to 4.81) in 2000 to 7.22 (95% CI: 7.07 to 7.37) in 2025. Among women, the AAMR rose from 2.79 (95% CI: 2.69 to 2.89) in 2000 to 5.03 (95% CI: 4.92 to 5.14) in 2025.

Men consistently exhibited higher mean AAMRs compared to women (mean AAMR for men: 6.16; 95% CI: 6.01 to 6.32; for women: 4.07; 95% CI: 3.96 to 4.18) throughout the study timeline.

From 2000 to 2025, the AAMR trend for both men and women significantly increased, [men: AAPC: 1.75 (95% CI: 1.58 to 1.96; p value = < 0.001); women: AAPC: 2.31 (95% CI: 2.15 to 2.51; p value = < 0.001)] (Supplemental Table 3).

By 2035, projections show male AAMR reaching 7.24 (95% CI: 4.89 to 10.72) and female AAMR reaching 5.10 (95% CI: 2.82 to 9.22) (Fig. 2) (Supplemental Table 9).

### Stratified by race/ethnicity

When stratified by race/ethnicity, mean AAMRs were highest among NH Black (7.38; 95% CI: 7.01 to 7.76), followed by NH White (5.05; 95% CI: 4.95 to 5.16), NH Asians/Pacific Islanders (3.09; 95% CI: 2.71 to 3.49), Hispanics (2.54; 95% CI: 2.29 to 2.79).

The AAMR increased across Hispanics (1.8 to 2.97), NH Asian (2.36 to 3.00), NH Blacks (6.59 to 7.74) and NH Whites (3.39 to 6.46) from 2000 to 2025.

Notably, the AAMR trend increased from 2000 to 2025 among Hispanics (AAPC: 1.78 (95% CI: 1.30 to 2.45; p value = < 0.001) and NH Whites (AAPC: 2.51 (95% CI: 2.32 to 2.74; p value = < 0.001), NH Black (AAPC: 0.53 (95% CI: 0.21 to 0.86; p value = < 0.001) and NH Asians/Pacific Islanders (AAPC: 0.62 (95% CI: 0.31 to 1.06; p value = < 0.001) (Supplemental Table 5).

**Fig. 3:**
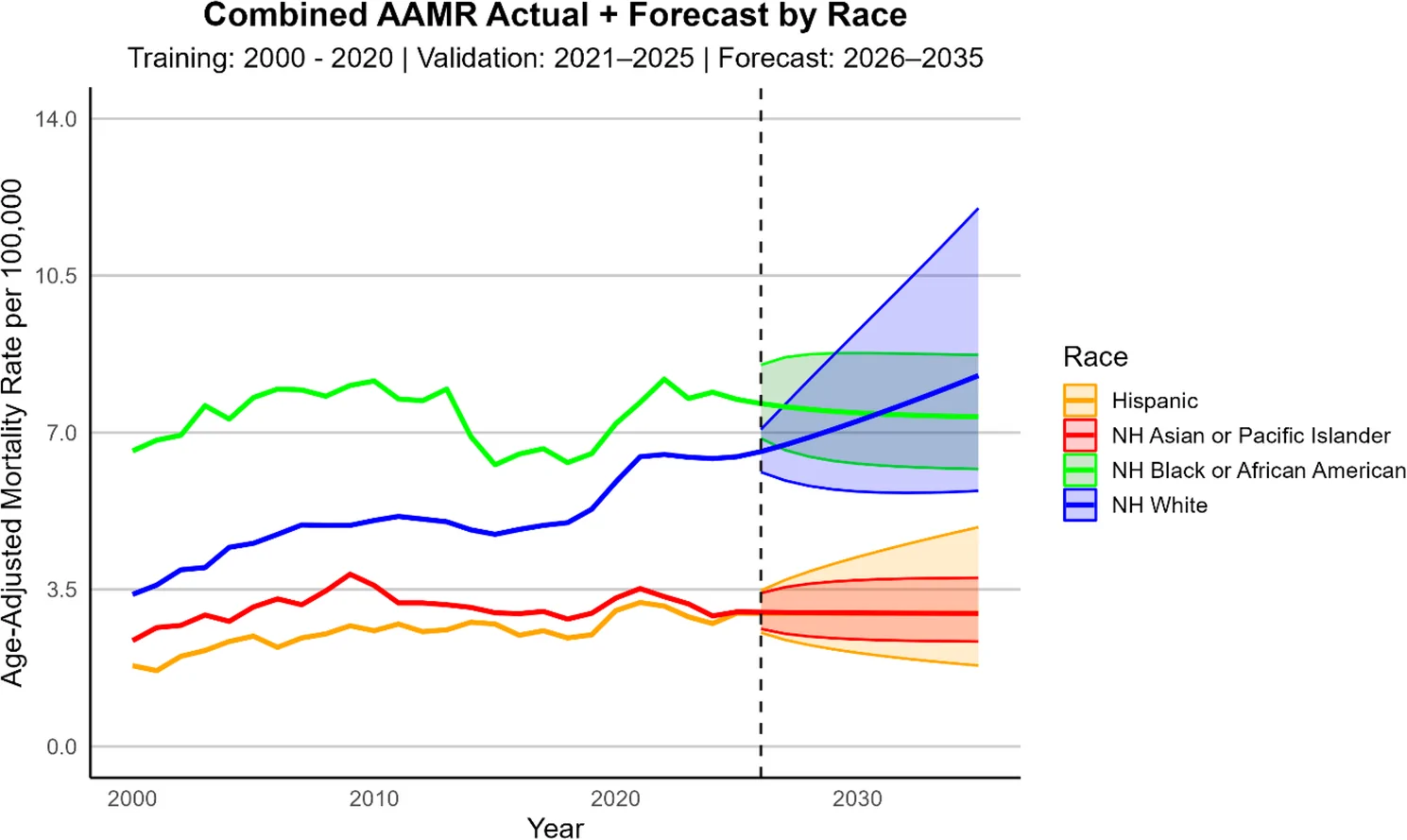
Lung cancer and co-listed hypertension AAMR stratified by race per 100,000 population

Projections for 2035 estimate, Hispanic AAMR at 2.97 (95% CI: 1.80 to 4.88), NH Asian or Pacific Islander at 2.96 (95% CI: 2.33 to 3.76), NH Black/African American at 7.34 (95% CI: 6.18 to 8.73), and NH White AAMR at 8.27 (95% CI: 5.69 to 12.00) (Fig. 3) (Supplemental Table 4) (Supplemental Table 8).

### Stratified by geographical region

#### Stratified by census region

From 2000 to 2025, the AAMR increased across all U.S. census regions. In the Northeast, the AAMR rose from 3.27 in 2000 to 4.04 in 2025; in the Midwest, from 3.82 to 6.25; in the South, from 3.56 to 7.84; and in the West, from 3.52 to 4.32.

Throughout the entire study period from 2000 to 2025, the highest mean AAMR was observed in the South (mean AAMR: 5.67; 95% CI: 5.51 to 5.83), followed by the Midwest (mean AAMR: 5.23; 95% CI: 5.03 to 5.43), the West (mean AAMR: 4.32; 95% CI: 4.13 to 4.51), and the Northeast (mean AAMR: 4.04; 95% CI: 3.85 to 4.22).

From 2000 to 2025, the AAMR trend remained stable with a slight rise in Northeast and West, while it had a significantly greater rise in the South and Midwest [Northeast: AAPC: 0.81 (95% CI: 0.51 to 1.10; p value = < 0.001); South: AAPC: 3.08 (95% CI: 2.85 to 3.42; p value = < 0.001); West: AAPC: 0.77 (95% CI: 0.53 to 1.05; p value = < 0.001); Midwest: AAPC: 1.72 (95% CI: 1.44 to 2.04; p value = < 0.001)] (Supplemental Table 6).

**Fig. 4:**
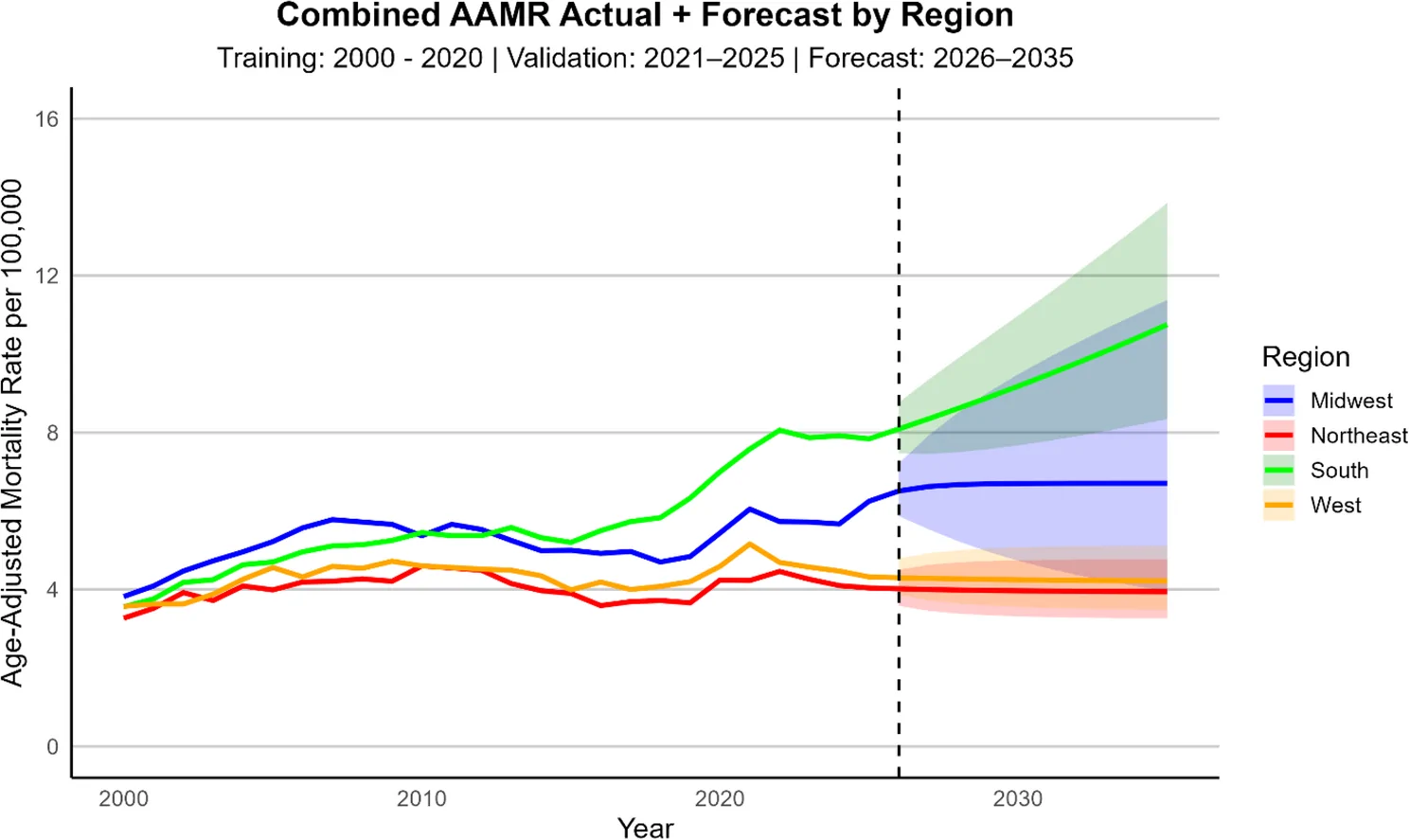
Lung Cancer and co-listed hypertension AAMR stratified by census per 100,000 population

Projections for 2035 estimate the Northeast AAMR at 3.94 (95% CI: 3.26 to 4.76), Midwest at 6.70 (95% CI: 3.95 to 11.37), South at 10.75 (95% CI: 8.34 to 13.85) and West at 4.21 (95% CI: 3.48 to 5.11) (Fig. 4) (Supplemental Table 8).

### Stratified by urbanization (2000–2020)

From 2000 to 2020, similar trends were observed across urban and rural areas. In urban areas, the AAMR increased from 3.53 in 2000 to 5.23 in 2020, similarly in rural areas it increased from 3.73 to 7.71.

Rural areas, however, showed higher AAMRs throughout the study timeline, with a mean AAMR of 5.73 for rural (95% CI: 5.49 to 5.97) and 4.5 for urban (95% CI: 4.41 to 4.61).

The AAMR trend for urban areas and rural areas showed a significant increase, with a greater rise observed in rural areas [Urban: AAPC: 1.91 (95% CI: 1.71 to 2.10; p value = < 0.001); Rural: AAPC: 3.72 (95% CI: 3.42 to 4.14; p value = < 0.001)] (Supplemental Table 5).

**Fig. 5:**
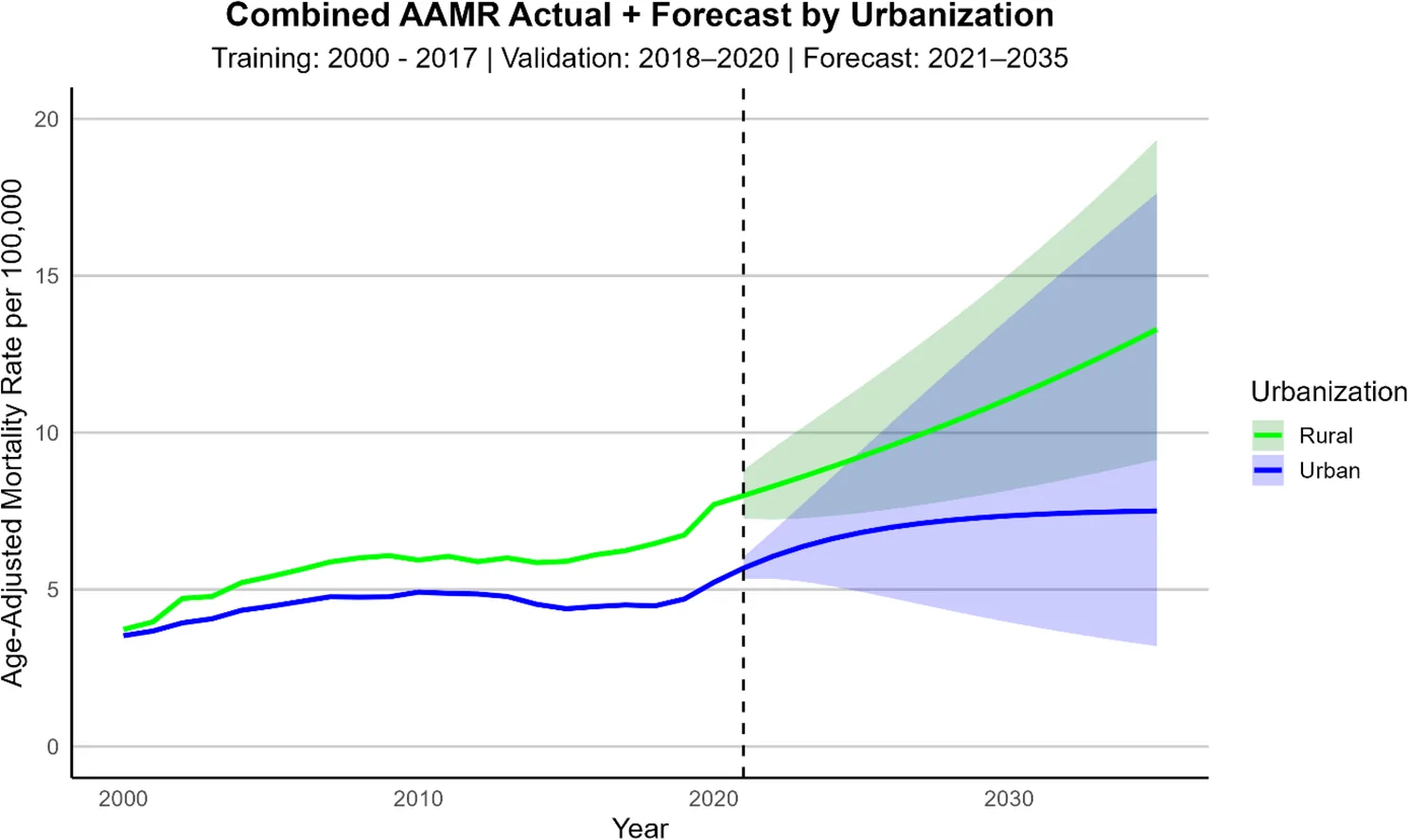
Lung cancer and co-listed hypertension AAMR stratified by urbanization per 100,000 population, 2000–2020

Projections show AAMR for urban areas reaching 7.50 (95% CI: 3.19 to 17.62) and for rural areas 13.29 (95% CI: 9.13 to 19.33) by 2035 (Fig. 5) (Supplemental Table 8).

### Stratified by states

**Fig. 6:**
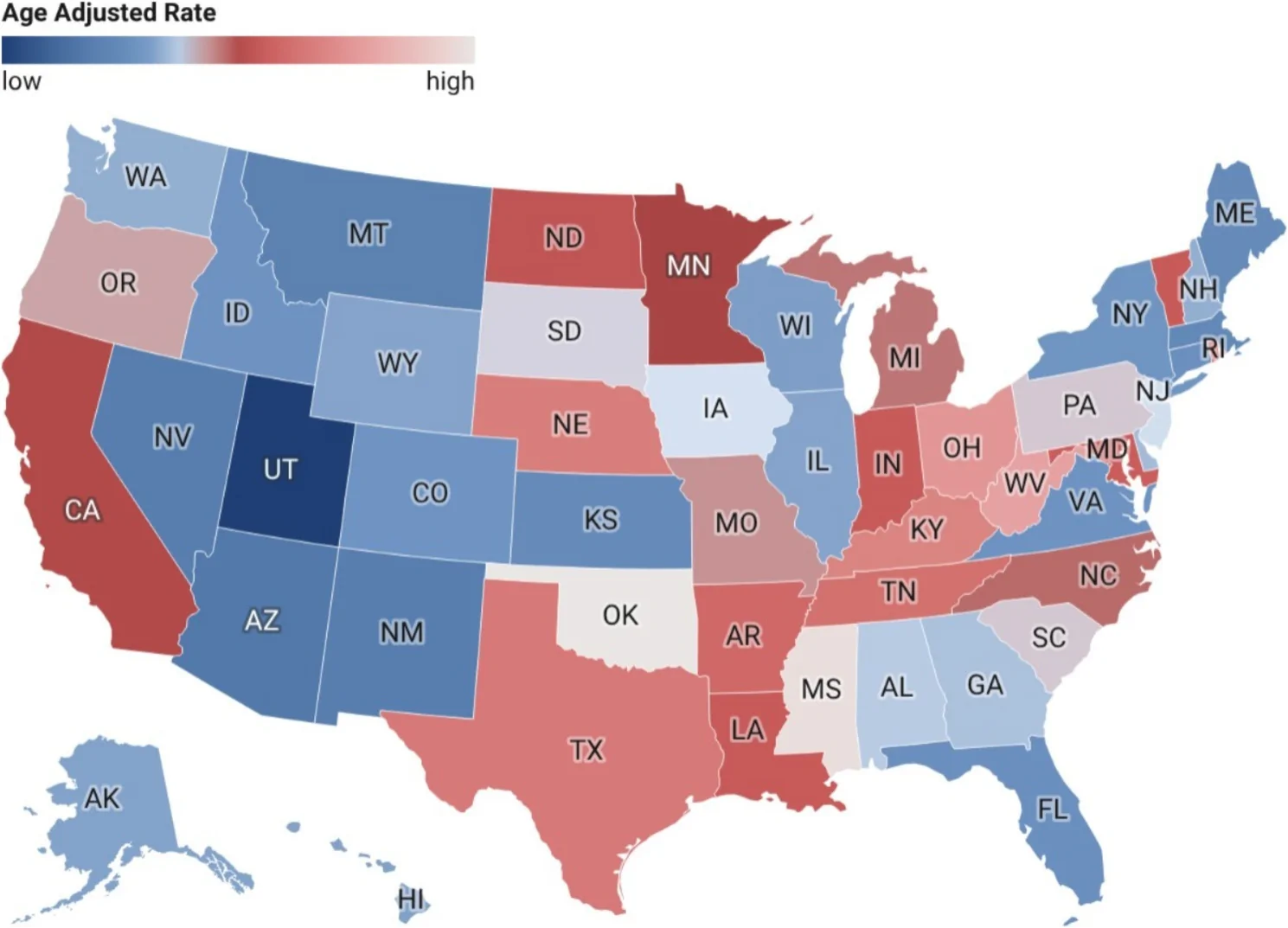
Lung cancer and co-listed hypertension AAMR stratified by state per 100,000 population

Disparities in AAMRs were observed among different states, with values ranging from as low as 1.04 (95% CI: 0.92–1.16) in Utah to as high as 9.58 (95% CI: 9.32–9.83) in Oklahoma. States States above the 90th percentile of mean AAMR included Mississippi, Nebraska, Kentucky, West Virginia, and Ohio, which exhibited several-fold higher AAMRs compared to states in the lower 10th percentile, including Utah, Nevada, Arizona, Kansas, and Maine (Fig. 6) (Supplemental Table 7).

## Discussion

**Fig. 7:**
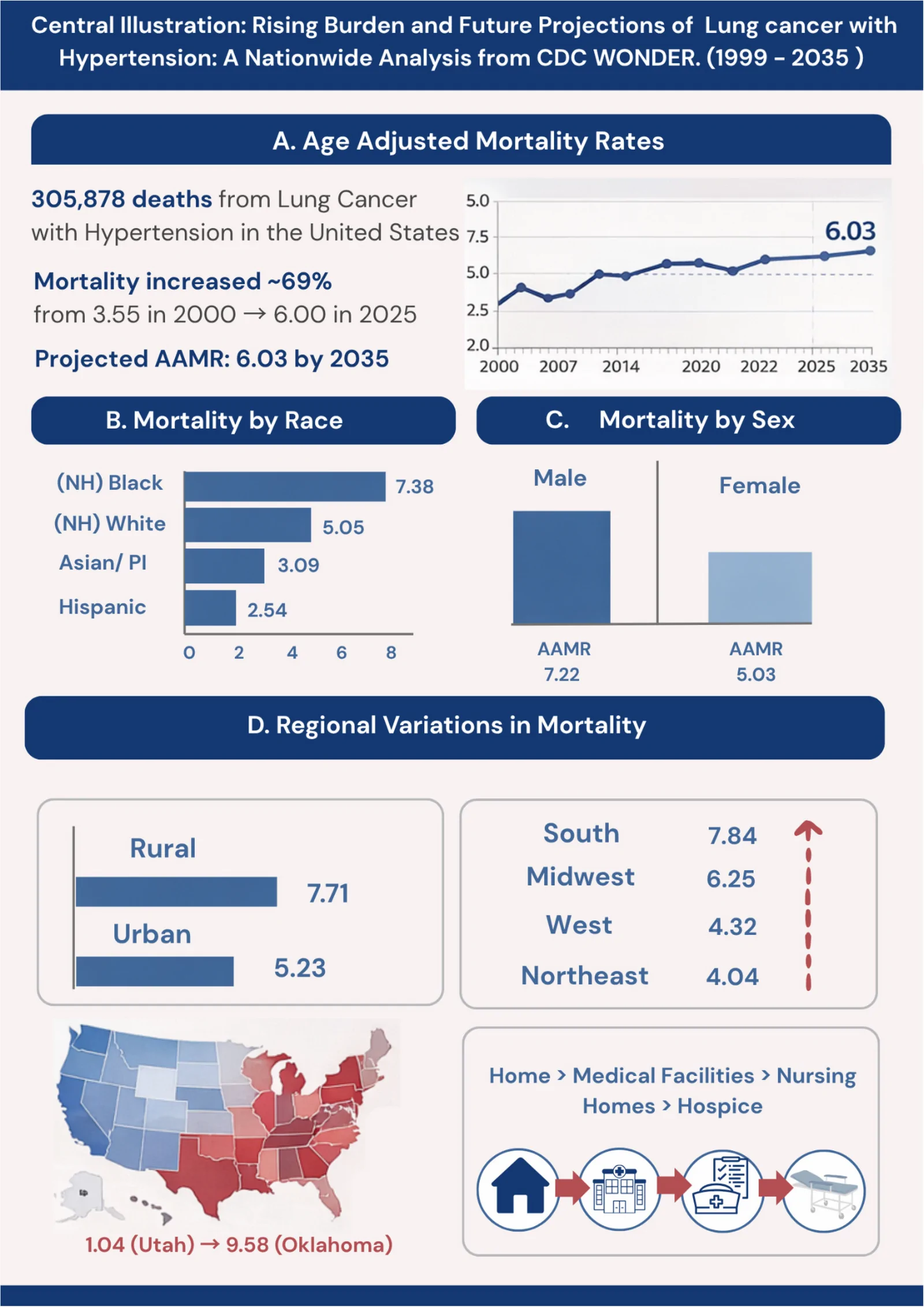
Graphical abstract: lung cancer and co-listed hypertension patients

Our study analyzed 25 years (2000–2025) of nationwide mortality data for LC and co-listed HTN among US adults aged 25 years and older. A total of 305,878 deaths were recorded, with most occurring in decedents’ homes, followed by medical facilities, nursing homes and hospice settings. Stratified analyses revealed consistent disparities across sex, race and geography. Our analysis demonstrated consistently higher mean AAMRs in men than women. By race and ethnicity, NH Black adults exhibited the highest mortality, followed by NH White and Asians/ Pacific Islanders, while Hispanics demonstrated comparatively lower mortality rates. Regionally, South demonstrated the highest AAMRs followed by the Midwest, West and then Northeast, with rural areas consistently exceeding urban areas. Projections for 2035 indicate that LC and co-listed HTN related mortality will remain relatively stable with a slight increase. Mortality rates are expected to remain higher in men. Although NH Black individuals currently demonstrate the highest mortality burden, the steeper temporal increase observed among NH White individuals suggests that by 2035 NH White individuals are expected to demonstrate the highest age-adjusted mortality rates, followed by NH Black individuals, while Hispanic and Asian/Pacific Islander populations are projected to maintain comparatively lower mortality. On a geographical basis, temporal patterns persist in projections, with the South and rural areas expected to exhibit the highest mortality by 2035. These findings highlight the need for sustained preventive and health system strategies to reduce future burden (Fig. 7).

The coexistence of LC and HTN represents a clinically recognized form of cardiometabolic–oncologic multimorbidity with overlapping risk factors and pathophysiological pathways as HTN is one of the most common comorbidities amongst cancer patients [18]. Specifically, LC has been reported to be associated with higher blood pressure levels, and prior studies suggest that increases in blood pressure may be linked with LC risk [9]. Several biological pathways may plausibly link LC with HTN including chronic inflammation, oxidative stress, endothelial dysfunction and metabolic dysregulation. Additionally, these conditions also share common environmental and behavioral exposures like tobacco usage, air pollution, dietary habits and occupational hazards which can significantly contribute to disease development and progression [9, 10]. With the growing prevalence of multimorbidity in the U.S population, the convergence of LC and HTN underscores a complex clinical entity driven by overlapping oncologic and cardiovascular pathways.

The temporal pattern observed across the study period may reflect evolving clinical practices, population level risk profiles, and broader healthcare system influences. The rise in mortality between 2000 and 2007 may be associated with increasing prevalence of HTN in early 2000s driven in part by rising obesity and the increase in comorbidities amongst hypertensive patients including cancer [19, 20]. These epidemiologic shifts, along with improved documentation of multiple contributing causes of death in national mortality databases, may also have influenced observed trends in comorbidity-stratified mortality estimates. The decline in mortality during 2007 to 2018 parallels the introduction of targeted therapies for LC during that timeline [21]. The sharp increase of LC related mortality during the SARS-CoV-2 pandemic may reflect disruptions in healthcare access, delayed cancer diagnosis and treatment, and broader changes in healthcare utilization [22–24]. Lastly, the decline in the post-pandemic era after 2022 likely reflects the restoration of routine hospital operations, patients resuming deferred care as healthcare services normalized and the resumption of cardiovascular care services [25, 26].

Men demonstrated higher mortality rates, which may be explained by both behavioral and biological factors. Epidemiological studies consistently report a higher prevalence of HTN among men compared to women. The sex-based differences observed in our study may similarly reflect underlying biological and hormonal influences [27]. However, given the descriptive nature of our study, these mechanisms remain speculative, and a causal relationship cannot be established. Additionally, behavioral risk profiles may further contribute to this disparity such as heightened exposure to smoking, alcohol consumption, and sedentary lifestyles prevalent among males [28, 29]. Together, these overlapping biological and behavioral mechanisms likely explain the greater mortality burden observed among men.

Racial and ethnic disparities in mortality were pronounced, with NH Black individuals exhibiting the highest burden. These differences are likely multifactorial, reflecting differences in baseline cardiometabolic risk profiles and structural determinants of health. Epidemiological studies highlight that NH Black individuals have the highest prevalence of HTN [30] and associated cardiovascular risk factors which may exacerbate disease severity and may contribute to worse outcomes in patients with LC. Additionally, evidence suggests that Black individuals may develop LC at younger ages and present with more advanced disease compared with other racial and ethnic groups. Beyond biological susceptibility, these disparities are partly influenced by structural and social determinants of health such as socioeconomic instability, unequal access to screening and early diagnosis and partial access to high quality treatment and surgical interventions as compared to other races [31–33].

The higher mortality burden observed in Southern states is consistent with prior literature demonstrating a disproportionate prevalence of cardiometabolic and LC associated risk factors in this region. This pattern may be related to historical higher smoking prevalence and weaker tobacco control policies in the region [34]. Furthermore, HTN is pre-dominant in residents of the South, likely attributed to various factors such as physical inactivity, poor dietary habits, lower income and education levels which are more common there [35]. Furthermore, rural populations consistently exhibited higher mortality than urban populations which similarly reflects the.

combined systemic and individual level barriers such as lack of awareness, limited healthcare services, delayed screening, lower provider density, and socioeconomic deprivation concentrated in rural Southern counties [36]. Collectively, these findings highlight that regional disease burden, state level healthcare capacity, and rural urban access gaps are central drivers of persistent geographic disparities in cardio-oncology mortality.

These observed temporal and subgroup patterns suggest how various factors contribute to the gender, racial and geographical related disparities observed in deaths associated with LC in hypertensive patients. Therefore, crucial preventive and management strategies like smoking cessation strategies, LC screening and optimized management of HTN risk factors through lifestyle and dietary changes can contribute to reducing future mortality burden and should be prioritized in both clinical practice and public health strategies [36].

### Limitations

This study has several limitations inherent to analyses based on death certificate–derived data. Estimates of LC and co-listed HTN related mortality data, rely on ICD-coded causes of death, which may be susceptible to misclassification or coding inaccuracies and could lead to potential underreporting or overreporting of disease involvement.

Additionally, ICD-10 coding cannot fully capture detailed clinical and demographic information including baseline patient profiles, smoking status, comorbid conditions, socioeconomic status, severity or duration of both diseases, timing of diagnosis, treatment approaches and adherence to it. The absence of these variables limits the ability to evaluate underlying risk factors or patient-level determinants of LC and co-listed HTN–related mortality. Moreover, urbanization classifications were not available in the dataset for years after 2020; therefore, analyses stratified by urban–rural status could not be conducted across the entire study period (2000–2025), which may limit interpretation of long-term urban–rural trends. Both ARIMA and Prophet models for time-series forecasting possess certain limitations. ARIMA relies on assumptions of linearity and stationarity, limiting its performance when dealing with nonlinear or long-term variations. In contrast, the Prophet model can oversimplify complex temporal patterns and is highly dependent on changepoint detection, additive seasonality assumptions, and careful parameter adjustment. Projections to 2035 should be interpreted cautiously, as future trends may shift with changes in health policies, coding practices, or population-level behaviors. Therefore, the findings are descriptive in nature and intended to generate hypotheses rather than provide definitive predictions. Despite these limitations, the use of a large, nationally representative dataset allows for a comprehensive assessment of long-term trends and disparities in LC with HTN–related mortality across the U.S.

## Conclusion

In conclusion, LC and co-listed HTN related mortality represents a growing public health concern in the US, characterized by persistent demographic and geographic disparities. Although recent trends demonstrate a post-pandemic decline, future projections based on historical patterns suggest that mortality may continue to rise through 2035 if current trends persist. These findings highlight the need for integrated cardiometabolic and oncologic prevention strategies, improved access to care and measures to optimize management of HTN and LC, particularly in vulnerable populations, to mitigate the ongoing and future burden of this comorbid condition.

## Supplementary Information

Supplementary Material 1.

## Data Availability

The datasets supporting the conclusions of this article are included within the article and its additional files.
