# Supplementary material for: Temporal trends and forecasted mortality involving lung cancer with co-listed hypertension in U.S. adults, 2000–2035
Source: Cardiooncology. 2026 Jun 20;12:112. doi: 10.1186/s40959-026-00524-9 (PMC13543550; doi:10.1186/s40959-026-00524-9)
Supplement: Supplementary file 1 [file 40959_2026_524_MOESM1_ESM.docx]

**Supplemental Table 1, Lung Cancer related mortalities in Adults with Hypertension, Stratified by Sex and Race, in the United States, 2000 to 2025**

| **Supplemental Table 1, Lung Cancer related mortalities in Adults with Hypertension, Stratified by Sex and Race, in the United States, 2000 to 2025** | | | | | | | | |
| --- | --- | --- | --- | --- | --- | --- | --- | --- |
| **Deaths** | | | | | | | | |
| **Year** | **Overall** | **Women** | **Men** | **NH Asian or Pacific Islander** | **NH Whites** | **Hispanics** | **NH Blacks** | **Population** |
| **2000** | 6364 | 2939 | 3425 | 94 | 5093 | 157 | 992 | 181984640 |
| **2001** | 6828 | 3257 | 3571 | 112 | 5457 | 164 | 1055 | 184305128 |
| **2002** | 7504 | 3558 | 3946 | 128 | 6038 | 201 | 1090 | 186208028 |
| **2003** | 7809 | 3714 | 4095 | 146 | 6187 | 219 | 1213 | 188090429 |
| **2004** | 8567 | 4047 | 4520 | 147 | 6927 | 249 | 1194 | 190205384 |
| **2005** | 8973 | 4252 | 4721 | 171 | 7175 | 278 | 1308 | 192551384 |
| **2006** | 9463 | 4546 | 4917 | 194 | 7570 | 266 | 1381 | 195019359 |
| **2007** | 9961 | 4662 | 5299 | 193 | 8004 | 303 | 1410 | 197403777 |
| **2008** | 10202 | 4782 | 5420 | 220 | 8163 | 329 | 1424 | 199795090 |
| **2009** | 10451 | 4850 | 5601 | 265 | 8253 | 366 | 1495 | 202107016 |
| **2010** | 10790 | 5077 | 5713 | 254 | 8531 | 370 | 1566 | 203891983 |
| **2011** | 11115 | 5163 | 5952 | 254 | 8813 | 423 | 1556 | 206592936 |
| **2012** | 11322 | 5296 | 6026 | 265 | 8975 | 416 | 1585 | 208826037 |
| **2013** | 11502 | 5272 | 6230 | 284 | 8996 | 441 | 1706 | 211085314 |
| **2014** | 11293 | 5095 | 6198 | 298 | 8877 | 500 | 1534 | 213809280 |
| **2015** | 11279 | 5144 | 6135 | 314 | 8866 | 529 | 1468 | 216553817 |
| **2016** | 11738 | 5359 | 6379 | 328 | 9254 | 502 | 1559 | 218641417 |
| **2017** | 12296 | 5599 | 6697 | 351 | 9647 | 557 | 1627 | 221447331 |
| **2018** | 12629 | 5704 | 6925 | 344 | 10015 | 539 | 1614 | 223311190 |
| **2019** | 13530 | 6118 | 7412 | 390 | 10716 | 580 | 1719 | 224981167 |
| **2020** | 15483 | 6999 | 8484 | 444 | 12201 | 744 | 1965 | 226635013 |
| **2021** | 16563 | 7642 | 8921 | 467 | 13035 | 796 | 2058 | 228238412 |
| **2022** | 17393 | 8168 | 9225 | 475 | 13649 | 796 | 2239 | 229508599 |
| **2023** | 17289 | 8030 | 9259 | 472 | 13622 | 780 | 2203 | 231529762 |
| **2024** | 17697 | 8180 | 9517 | 467 | 13895 | 780 | 2310 | 235615087 |
| **2025** | 17837 | 8263 | 9574 | 481 | 14001 | 850 | 2273 | 235615087 |
| **Total** | **305878** | **141716** | **164162** | **7558** | **241960** | **12135** | **41544** | **5453952667** |

**Supplemental Table 2, Lung Cancer related Mortality, Stratified by Place of Death, in Adults with Hypertension in the United States, 2000 to 2025**

| **Supplemental Table 2, Lung Cancer related Mortality, Stratified by Place of Death, in Adults with Hypertension in the United States, 2000 to 2025** | | | | |
| --- | --- | --- | --- | --- |
| **Deaths** | | | | |
| **Year** | **Medical Facility** | **Nursing Home/Long-term Care Facility** | **Hospice Facility** | **Decedent's Home** |
| **2000** | 2479 | 1364 | - | 2207 |
| **2001** | 2613 | 1464 | - | 2402 |
| **2002** | 2780 | 1640 | - | 2677 |
| **2003** | 2813 | 1700 | 22 | 2816 |
| **2004** | 3026 | 1868 | 56 | 3095 |
| **2005** | 3090 | 1904 | 173 | 3318 |
| **2006** | 3162 | 2051 | 214 | 3494 |
| **2007** | 3340 | 2037 | 433 | 3621 |
| **2008** | 3201 | 2111 | 509 | 3724 |
| **2009** | 3140 | 2104 | 539 | 3897 |
| **2010** | 3211 | 2194 | 635 | 4144 |
| **2011** | 3353 | 2209 | 742 | 4222 |
| **2012** | 3253 | 2124 | 832 | 4527 |
| **2013** | 3148 | 2195 | 808 | 4717 |
| **2014** | 2989 | 2061 | 958 | 4815 |
| **2015** | 3039 | 2035 | 1045 | 4702 |
| **2016** | 3153 | 2068 | 1135 | 4931 |
| **2017** | 3182 | 2082 | 1267 | 5330 |
| **2018** | 3266 | 2104 | 1372 | 5380 |
| **2019** | 3485 | 2180 | 1463 | 5818 |
| **2020** | 3838 | 1850 | 1373 | 7712 |
| **2021** | 4180 | 1924 | 1601 | 8134 |
| **2022** | 4462 | 2033 | 1870 | 8236 |
| **2023** | 4112 | 2194 | 2004 | 8255 |
| **2024** | 4383 | 2276 | 1907 | 8349 |
| **2025** | 4432 | 2329 | 1906 | 8444 |
| **Total** | **87130** | **52101** | **22864** | **128967** |

**Supplemental Table 3, Overall and Sex‐Stratified Lung Cancer related Age-Adjusted Mortality Rates per 100,000, in Adults with Hypertension in the United States, 2000 to 2025**

| **Supplemental Table 3, Overall and Sex‐Stratified Lung Cancer related Age-Adjusted Mortality Rates per 100,000, in Adults with Hypertension in the United States, 2000 to 2025** | | | |
| --- | --- | --- | --- |
| **Age-Adjusted Rate (95% CI)** | | | |
| **Year** | **Men** | **Women** | **Overall** |
| **2000** | 4.65 (4.49 - 4.81) | 2.79 (2.69 - 2.89) | 3.55 (3.46 - 3.64) |
| **2001** | 4.74 (4.58 - 4.90) | 3.03 (2.93 - 3.14) | 3.75 (3.66 - 3.84) |
| **2002** | 5.16 (5.00 - 5.33) | 3.31 (3.20 - 3.42) | 4.07 (3.97 - 4.16) |
| **2003** | 5.29 (5.13 - 5.46) | 3.40 (3.29 - 3.51) | 4.18 (4.09 - 4.27) |
| **2004** | 5.73 (5.56 - 5.90) | 3.65 (3.53 - 3.76) | 4.52 (4.43 - 4.62) |
| **2005** | 5.82 (5.65 - 5.99) | 3.80 (3.68 - 3.91) | 4.65 (4.56 - 4.75) |
| **2006** | 5.92 (5.76 - 6.09) | 4.00 (3.89 - 4.12) | 4.81 (4.71 - 4.90) |
| **2007** | 6.22 (6.05 - 6.39) | 4.03 (3.91 - 4.15) | 4.96 (4.86 - 5.05) |
| **2008** | 6.24 (6.07 - 6.41) | 4.07 (3.95 - 4.18) | 4.97 (4.87 - 5.06) |
| **2009** | 6.32 (6.15 - 6.48) | 4.08 (3.96 - 4.19) | 5.01 (4.91 - 5.10) |
| **2010** | 6.29 (6.13 - 6.46) | 4.18 (4.07 - 4.30) | 5.08 (4.99 - 5.18) |
| **2011** | 6.35 (6.19 - 6.51) | 4.14 (4.03 - 4.26) | 5.10 (5.01 - 5.20) |
| **2012** | 6.23 (6.07 - 6.39) | 4.18 (4.06 - 4.29) | 5.04 (4.95 - 5.14) |
| **2013** | 6.23 (6.07 - 6.39) | 4.05 (3.94 - 4.16) | 5.01 (4.92 - 5.11) |
| **2014** | 5.97 (5.82 - 6.13) | 3.86 (3.75 - 3.97) | 4.75 (4.66 - 4.84) |
| **2015** | 5.78 (5.63 - 5.93) | 3.77 (3.66 - 3.87) | 4.66 (4.58 - 4.75) |
| **2016** | 5.87 (5.72 - 6.02) | 3.85 (3.75 - 3.96) | 4.74 (4.65 - 4.83) |
| **2017** | 6.00 (5.85 - 6.15) | 3.89 (3.79 - 3.99) | 4.81 (4.72 - 4.90) |
| **2018** | 5.98 (5.84 - 6.13) | 3.88 (3.78 - 3.99) | 4.81 (4.72 - 4.89) |
| **2019** | 6.25 (6.10 - 6.39) | 4.12 (4.01 - 4.22) | 5.03 (4.95 - 5.12) |
| **2020** | 6.97 (6.82 - 7.12) | 4.54 (4.43 - 4.65) | 5.64 (5.55 - 5.73) |
| **2021** | 7.40 (7.24 - 7.55) | 5.07 (4.95 - 5.19) | 6.12 (6.03 - 6.22) |
| **2022** | 7.36 (7.21 - 7.51) | 5.18 (5.07 - 5.30) | 6.15 (6.06 - 6.25) |
| **2023** | 7.21 (7.07 - 7.37) | 5.05 (4.94 - 5.16) | 6.03 (5.94 - 6.12) |
| **2024** | 7.19 (7.04 - 7.34) | 5.00 (4.89 - 5.11) | 5.98 (5.89 - 6.07) |
| **2025** | 7.22 (7.07 - 7.37) | 5.03 (4.92 - 5.14) | 6.00 (5.91 - 6.09) |
| **Total** | **6.16 (6.01 - 6.32)** | **4.07 (3.96 - 4.18)** | **4.97 (4.88 - 5.07)** |

**Supplemental Table 4, Race‐Stratified Lung Cancer related Age-Adjusted Mortality Rates per 100,000 in Adults with Hypertension in the United States, 2000 to 2025**

| **Supplemental Table 4, Race‐Stratified Lung Cancer related Age-Adjusted Mortality Rates per 100,000 in Adults with Hypertension in the United States, 2000 to 2025** | | | | |
| --- | --- | --- | --- | --- |
| **Age-Adjusted Rate (95% CI)** | | | | |
| **Year** | **NH Asian or Pacific Islander** | **NH Black** | **Hispanic** | **NH White** |
| **2000** | 2.36 (1.89 - 2.90) | 6.59 (6.17 - 7.00) | 1.80 (1.51 - 2.09) | 3.39 (3.30 - 3.49) |
| **2001** | 2.65 (2.14 - 3.15) | 6.83 (6.41 - 7.25) | 1.69 (1.42 - 1.95) | 3.60 (3.51 - 3.70) |
| **2002** | 2.70 (2.22 - 3.18) | 6.94 (6.52 - 7.36) | 2.01 (1.72 - 2.30) | 3.94 (3.84 - 4.04) |
| **2003** | 2.93 (2.44 - 3.41) | 7.60 (7.17 - 8.04) | 2.14 (1.84 - 2.43) | 3.99 (3.89 - 4.09) |
| **2004** | 2.79 (2.33 - 3.26) | 7.30 (6.87 - 7.72) | 2.34 (2.05 - 2.64) | 4.44 (4.34 - 4.55) |
| **2005** | 3.11 (2.63 - 3.58) | 7.78 (7.35 - 8.21) | 2.46 (2.17 - 2.76) | 4.53 (4.43 - 4.64) |
| **2006** | 3.29 (2.82 - 3.77) | 7.97 (7.54 - 8.40) | 2.21 (1.94 - 2.49) | 4.73 (4.62 - 4.83) |
| **2007** | 3.16 (2.70 - 3.61) | 7.95 (7.53 - 8.38) | 2.42 (2.14 - 2.70) | 4.94 (4.83 - 5.04) |
| **2008** | 3.47 (3.01 - 3.94) | 7.81 (7.40 - 8.23) | 2.51 (2.24 - 2.79) | 4.93 (4.82 - 5.03) |
| **2009** | 3.84 (3.36 - 4.31) | 8.05 (7.63 - 8.47) | 2.69 (2.40 - 2.97) | 4.93 (4.82 - 5.03) |
| **2010** | 3.59 (3.14 - 4.03) | 8.15 (7.74 - 8.57) | 2.58 (2.31 - 2.85) | 5.04 (4.93 - 5.14) |
| **2011** | 3.20 (2.80 - 3.60) | 7.75 (7.35 - 8.15) | 2.73 (2.46 - 3.00) | 5.13 (5.02 - 5.24) |
| **2012** | 3.20 (2.81 - 3.59) | 7.71 (7.32 - 8.10) | 2.56 (2.30 - 2.81) | 5.07 (4.97 - 5.18) |
| **2013** | 3.16 (2.79 - 3.54) | 7.97 (7.58 - 8.36) | 2.60 (2.35 - 2.85) | 5.01 (4.90 - 5.11) |
| **2014** | 3.10 (2.74 - 3.46) | 6.91 (6.55 - 7.27) | 2.77 (2.52 - 3.01) | 4.83 (4.73 - 4.93) |
| **2015** | 2.98 (2.64 - 3.31) | 6.28 (5.95 - 6.61) | 2.73 (2.49 - 2.96) | 4.73 (4.63 - 4.84) |
| **2016** | 2.96 (2.64 - 3.29) | 6.52 (6.19 - 6.86) | 2.48 (2.26 - 2.70) | 4.84 (4.74 - 4.94) |
| **2017** | 3.01 (2.69 - 3.33) | 6.64 (6.31 - 6.98) | 2.58 (2.36 - 2.80) | 4.93 (4.83 - 5.03) |
| **2018** | 2.84 (2.53 - 3.14) | 6.33 (6.01 - 6.65) | 2.42 (2.21 - 2.63) | 4.99 (4.90 - 5.09) |
| **2019** | 2.97 (2.67 - 3.27) | 6.53 (6.21 - 6.85) | 2.49 (2.28 - 2.70) | 5.29 (5.19 - 5.39) |
| **2020** | 3.31 (2.99 - 3.62) | 7.20 (6.87 - 7.53) | 3.03 (2.81 - 3.26) | 5.90 (5.79 - 6.00) |
| **2021** | 3.52 (3.20 - 3.85) | 7.67 (7.33 - 8.02) | 3.21 (2.98 - 3.45) | 6.46 (6.35 - 6.58) |
| **2022** | 3.34 (3.04 - 3.66) | 8.19 (7.84 - 8.54) | 3.13 (2.91 - 3.36) | 6.51 (6.40 - 6.62) |
| **2023** | 3.18 (2.90 - 3.49) | 7.76 (7.43 - 8.10) | 2.89 (2.69 - 3.11) | 6.45 (6.34 - 6.56) |
| **2024** | 2.91 (2.65 - 3.19) | 7.90 (7.57 - 8.24) | 2.74 (2.54 - 2.94) | 6.42 (6.31 - 6.53) |
| **2025** | 3.00 (2.74 - 3.28) | 7.74 (7.42 - 8.07) | 2.97 (2.77 - 3.18) | 6.46 (6.35 - 6.57) |
| **Total** | **3.09 (2.71 - 3.49)** | **7.38 (7.01 - 7.76)** | **2.54 (2.77 - 3.18)** | **5.05 (4.95 - 5.16)** |
| NH = Non-Hispanic | | | | |

**Supplemental Table 5, Lung Cancer related Age-Adjusted Mortality Rates per 100,000, Stratified by Urban-Rural Classification, in Adults with Hypertension in the United States, 2000 to 2020**

| **Supplemental Table 5, Lung Cancer related Age-Adjusted Mortality Rates per 100,000, Stratified by Urban-Rural Classification, in Adults with Hypertension in the United States, 2000 to 2020** | | |
| --- | --- | --- |
| **Age-Adjusted Rate (95% CI)** | | |
| **Year** | **Urban** | **Rural** |
| **2000** | 3.53 (3.43 - 3.63) | 3.73 (3.52 - 3.94) |
| **2001** | 3.68 (3.58 - 3.78) | 3.97 (3.75 - 4.18) |
| **2002** | 3.94 (3.84 - 4.04) | 4.72 (4.49 - 4.95) |
| **2003** | 4.07 (3.97 - 4.17) | 4.78 (4.55 - 5.01) |
| **2004** | 4.34 (4.23 - 4.44) | 5.22 (4.98 - 5.46) |
| **2005** | 4.47 (4.37 - 4.58) | 5.42 (5.18 - 5.67) |
| **2006** | 4.62 (4.52 - 4.73) | 5.64 (5.39 - 5.88) |
| **2007** | 4.77 (4.66 - 4.88) | 5.88 (5.63 - 6.13) |
| **2008** | 4.76 (4.65 - 4.86) | 6.01 (5.76 - 6.26) |
| **2009** | 4.77 (4.67 - 4.88) | 6.08 (5.82 - 6.33) |
| **2010** | 4.92 (4.82 - 5.03) | 5.94 (5.69 - 6.18) |
| **2011** | 4.88 (4.78 - 4.99) | 6.06 (5.81 - 6.30) |
| **2012** | 4.86 (4.76 - 4.96) | 5.89 (5.65 - 6.13) |
| **2013** | 4.78 (4.68 - 4.88) | 6.01 (5.77 - 6.25) |
| **2014** | 4.53 (4.43 - 4.63) | 5.86 (5.63 - 6.10) |
| **2015** | 4.39 (4.30 - 4.48) | 5.90 (5.67 - 6.14) |
| **2016** | 4.46 (4.36 - 4.55) | 6.11 (5.87 - 6.35) |
| **2017** | 4.51 (4.42 - 4.60) | 6.24 (6.00 - 6.47) |
| **2018** | 4.48 (4.39 - 4.57) | 6.47 (6.23 - 6.71) |
| **2019** | 4.70 (4.61 - 4.80) | 6.74 (6.50 - 6.98) |
| **2020** | 5.23 (5.14 - 5.33) | 7.71 (7.45 - 7.97) |
| **Total** | **4.50 (4.41 - 4.61)** | **5.73 (5.49 - 5.97)** |
| The data for urbanization is only available till 2020 in the CDC Wonder Database. | | |
|  |  |  |

**Supplemental Table 6, Lung Cancer related Age-Adjusted Mortality Rates per 100,000, Stratified by Census Region, in Adults with Hypertension in the United States, 2000 to 2025**

| **Supplemental Table 6, Lung Cancer related Age-Adjusted Mortality Rates per 100,000, Stratified by Census Region, in Adults with Hypertension in the United States, 2000 to 2025** | | | | |
| --- | --- | --- | --- | --- |
| **Age-Adjusted Rate (95% CI)** | | | | |
| **Year** | **Northeast** | **Midwest** | **South** | **West** |
| **2000** | 3.27 (3.08 - 3.45) | 3.82 (3.63 - 4.01) | 3.56 (3.41 - 3.70) | 3.57 (3.37 - 3.76) |
| **2001** | 3.52 (3.33 - 3.71) | 4.09 (3.89 - 4.28) | 3.76 (3.61 - 3.91) | 3.63 (3.43 - 3.82) |
| **2002** | 3.92 (3.72 - 4.12) | 4.47 (4.26 - 4.67) | 4.18 (4.02 - 4.33) | 3.63 (3.44 - 3.83) |
| **2003** | 3.72 (3.53 - 3.91) | 4.73 (4.53 - 4.94) | 4.25 (4.09 - 4.41) | 3.88 (3.68 - 4.07) |
| **2004** | 4.09 (3.89 - 4.29) | 4.96 (4.75 - 5.17) | 4.63 (4.47 - 4.79) | 4.26 (4.05 - 4.46) |
| **2005** | 3.99 (3.79 - 4.18) | 5.22 (5.00 - 5.43) | 4.70 (4.53 - 4.86) | 4.56 (4.35 - 4.78) |
| **2006** | 4.19 (3.98 - 4.39) | 5.57 (5.35 - 5.79) | 4.96 (4.80 - 5.13) | 4.32 (4.12 - 4.52) |
| **2007** | 4.21 (4.01 - 4.42) | 5.78 (5.56 - 6.00) | 5.11 (4.95 - 5.28) | 4.59 (4.38 - 4.79) |
| **2008** | 4.27 (4.07 - 4.48) | 5.72 (5.50 - 5.93) | 5.14 (4.98 - 5.30) | 4.54 (4.34 - 4.75) |
| **2009** | 4.21 (4.01 - 4.41) | 5.66 (5.44 - 5.87) | 5.25 (5.09 - 5.42) | 4.72 (4.51 - 4.92) |
| **2010** | 4.60 (4.39 - 4.81) | 5.37 (5.16 - 5.58) | 5.45 (5.28 - 5.61) | 4.60 (4.40 - 4.80) |
| **2011** | 4.55 (4.35 - 4.76) | 5.66 (5.44 - 5.87) | 5.37 (5.20 - 5.53) | 4.56 (4.36 - 4.75) |
| **2012** | 4.49 (4.29 - 4.69) | 5.53 (5.32 - 5.74) | 5.37 (5.21 - 5.53) | 4.52 (4.33 - 4.71) |
| **2013** | 4.15 (3.95 - 4.34) | 5.25 (5.05 - 5.45) | 5.58 (5.42 - 5.74) | 4.49 (4.30 - 4.68) |
| **2014** | 3.97 (3.78 - 4.16) | 4.99 (4.80 - 5.19) | 5.32 (5.17 - 5.47) | 4.35 (4.16 - 4.53) |
| **2015** | 3.90 (3.72 - 4.09) | 5.00 (4.81 - 5.19) | 5.20 (5.05 - 5.35) | 3.99 (3.82 - 4.16) |
| **2016** | 3.59 (3.41 - 3.76) | 4.92 (4.73 - 5.12) | 5.50 (5.35 - 5.65) | 4.19 (4.01 - 4.36) |
| **2017** | 3.69 (3.52 - 3.87) | 4.97 (4.78 - 5.16) | 5.73 (5.58 - 5.88) | 4.00 (3.83 - 4.16) |
| **2018** | 3.72 (3.54 - 3.89) | 4.70 (4.52 - 4.88) | 5.83 (5.68 - 5.98) | 4.08 (3.91 - 4.24) |
| **2019** | 3.66 (3.49 - 3.83) | 4.84 (4.66 - 5.02) | 6.33 (6.17 - 6.49) | 4.20 (4.03 - 4.37) |
| **2020** | 4.24 (4.06 - 4.42) | 5.44 (5.25 - 5.63) | 7.00 (6.84 - 7.16) | 4.59 (4.42 - 4.76) |
| **2021** | 4.23 (4.05 - 4.41) | 6.05 (5.85 - 6.26) | 7.58 (7.42 - 7.76) | 5.16 (4.98 - 5.35) |
| **2022** | 4.46 (4.28 - 4.65) | 5.73 (5.54 - 5.93) | 8.06 (7.89 - 8.23) | 4.69 (4.52 - 4.87) |
| **2023** | 4.26 (4.08 - 4.44) | 5.72 (5.53 - 5.92) | 7.87 (7.70 - 8.04) | 4.57 (4.40 - 4.74) |
| **2024** | 4.10 (3.93 - 4.27) | 5.67 (5.48 - 5.86) | 7.92 (7.75 - 8.08) | 4.47 (4.31 - 4.64) |
| **2025** | 4.04 (3.87 - 4.21) | 6.25 (6.05 - 6.45) | 7.84 (7.67 - 8.00) | 4.32 (4.16 - 4.48) |
| **Total** | **4.04 (3.85 - 4.22)** | **5.23 (5.03 - 5.43)** | **5.67 (5.51 - 5.83)** | **4.32 (4.13 - 4.51)** |

**Supplemental Table 7, Lung Cancer related Age-Adjusted Mortality Rates per 100,000, Stratified by States, in Adults with Hypertension in the United States, 2000 to 2025**

| **Supplemental Table 7, Lung Cancer related Age-Adjusted Mortality Rates per 100,000, Stratified by States, in Adults with Hypertension in the United States, 2000 to 2025** | |
| --- | --- |
| **State** | **Age-Adjusted Rate (95% CI)** |
| Alabama | 4.24 (4.09 - 4.39) |
| Alaska | 3.80 (3.29 - 4.31) |
| Arizona | 2.43 (2.33 - 2.53) |
| Arkansas | 6.04 (5.82 - 6.27) |
| California | 5.24 (5.18 - 5.31) |
| Colorado | 3.58 (3.43 - 3.73) |
| Connecticut | 3.42 (3.27 - 3.57) |
| Delaware | 4.09 (3.76 - 4.42) |
| District of Columbia | 6.18 (5.63 - 6.73) |
| Florida | 3.43 (3.37 - 3.49) |
| Georgia | 4.14 (4.02 - 4.26) |
| Hawaii | 3.77 (3.51 - 4.02) |
| Idaho | 3.56 (3.31 - 3.81) |
| Illinois | 3.81 (3.73 - 3.90) |
| Indiana | 5.68 (5.53 - 5.83) |
| Iowa | 4.47 (4.29 - 4.66) |
| Kansas | 3.25 (3.08 - 3.42) |
| Kentucky | 6.84 (6.63 - 7.04) |
| Louisiana | 5.68 (5.50 - 5.87) |
| Maine | 3.25 (3.01 - 3.48) |
| Maryland | 5.72 (5.56 - 5.89) |
| Massachusetts | 3.13 (3.02 - 3.24) |
| Michigan | 4.89 (4.78 - 5.00) |
| Minnesota | 5.10 (4.94 - 5.26) |
| Mississippi | 9.34 (9.05 - 9.63) |
| Missouri | 4.78 (4.64 - 4.92) |
| Montana | 2.84 (2.58 - 3.10) |
| Nebraska | 6.65 (6.35 - 6.95) |
| Nevada | 2.65 (2.48 - 2.83) |
| New Hampshire | 3.93 (3.66 - 4.20) |
| New Jersey | 4.43 (4.32 - 4.54) |
| New Mexico | 2.63 (2.45 - 2.82) |
| New York | 3.62 (3.55 - 3.68) |
| North Carolina | 4.93 (4.81 - 5.05) |
| North Dakota | 5.53 (5.09 - 5.97) |
| Ohio | 7.33 (7.21 - 7.46) |
| Oklahoma | 9.58 (9.32 - 9.83) |
| Oregon | 4.71 (4.54 - 4.89) |
| Pennsylvania | 4.57 (4.49 - 4.66) |
| Rhode Island | 6.25 (5.88 - 6.63) |
| South Carolina | 4.57 (4.42 - 4.73) |
| South Dakota | 4.53 (4.16 - 4.89) |
| Tennessee | 6.29 (6.14 - 6.45) |
| Texas | 6.52 (6.43 - 6.61) |
| Utah | 1.04 (0.92 - 1.16) |
| Vermont | 5.68 (5.21 - 6.14) |
| Virginia | 3.54 (3.43 - 3.65) |
| Washington | 3.93 (3.80 - 4.06) |
| West Virginia | 7.66 (7.36 - 7.96) |
| Wisconsin | 3.74 (3.61 - 3.86) |
| Wyoming | 3.81 (3.37 - 4.24) |

**Supplemental Table 8, Lung Cancer with Hypertension related Forecast Age-Adjusted Mortality Rates, Stratified by Overall, Sex, Race, Age, Census, Urbanization in Adults in the United States, 2026 to 2035**

| **Supplemental Table 8, Lung Cancer with Hypertension related Forecast Age-Adjusted Mortality Rates, Stratified by Overall, Sex, Race, Age, Census, Urbanization in Adults in the United States, 2026 to 2035** | | | |
| --- | --- | --- | --- |
| **Stratification** | **Year** | **Forecast Value (LCI - UCI)** | **Model** |
| Female | 2026 | 5.05 (4.72 - 5.40) | ARIMA |
| Female | 2027 | 5.06 (4.42 - 5.79) | ARIMA |
| Female | 2028 | 5.07 (4.14 - 6.21) | ARIMA |
| Female | 2029 | 5.08 (3.89 - 6.64) | ARIMA |
| Female | 2030 | 5.09 (3.65 - 7.08) | ARIMA |
| Female | 2031 | 5.09 (3.45 - 7.52) | ARIMA |
| Female | 2032 | 5.09 (3.26 - 7.95) | ARIMA |
| Female | 2033 | 5.10 (3.10 - 8.38) | ARIMA |
| Female | 2034 | 5.10 (2.95 - 8.80) | ARIMA |
| Female | 2035 | 5.10 (2.82 - 9.22) | ARIMA |
| Male | 2026 | 7.23 (6.75 - 7.74) | ARIMA |
| Male | 2027 | 7.24 (6.40 - 8.19) | ARIMA |
| Male | 2028 | 7.24 (6.10 - 8.60) | ARIMA |
| Male | 2029 | 7.24 (5.85 - 8.97) | ARIMA |
| Male | 2030 | 7.24 (5.64 - 9.31) | ARIMA |
| Male | 2031 | 7.24 (5.45 - 9.62) | ARIMA |
| Male | 2032 | 7.24 (5.29 - 9.92) | ARIMA |
| Male | 2033 | 7.24 (5.14 - 10.20) | ARIMA |
| Male | 2034 | 7.24 (5.01 - 10.47) | ARIMA |
| Male | 2035 | 7.24 (4.89 - 10.72) | ARIMA |
| Overall | 2026 | 6.01 (5.63 - 6.41) | ARIMA |
| Overall | 2027 | 6.02 (5.31 - 6.81) | ARIMA |
| Overall | 2028 | 6.02 (5.02 - 7.23) | ARIMA |
| Overall | 2029 | 6.03 (4.75 - 7.65) | ARIMA |
| Overall | 2030 | 6.03 (4.51 - 8.05) | ARIMA |
| Overall | 2031 | 6.03 (4.30 - 8.45) | ARIMA |
| Overall | 2032 | 6.03 (4.12 - 8.83) | ARIMA |
| Overall | 2033 | 6.03 (3.95 - 9.20) | ARIMA |
| Overall | 2034 | 6.03 (3.81 - 9.56) | ARIMA |
| Overall | 2035 | 6.03 (3.67 - 9.91) | ARIMA |
| Hispanic | 2026 | 2.97 (2.53 - 3.47) | ARIMA |
| Hispanic | 2027 | 2.97 (2.37 - 3.71) | ARIMA |
| Hispanic | 2028 | 2.97 (2.26 - 3.90) | ARIMA |
| Hispanic | 2029 | 2.97 (2.16 - 4.07) | ARIMA |
| Hispanic | 2030 | 2.97 (2.08 - 4.22) | ARIMA |
| Hispanic | 2031 | 2.97 (2.01 - 4.36) | ARIMA |
| Hispanic | 2032 | 2.97 (1.95 - 4.50) | ARIMA |
| Hispanic | 2033 | 2.97 (1.90 - 4.63) | ARIMA |
| Hispanic | 2034 | 2.97 (1.85 - 4.76) | ARIMA |
| Hispanic | 2035 | 2.97 (1.80 - 4.88) | ARIMA |
| NH Asian or Pacific Islander | 2026 | 2.99 (2.62 - 3.41) | ARIMA |
| NH Asian or Pacific Islander | 2027 | 2.98 (2.51 - 3.55) | ARIMA |
| NH Asian or Pacific Islander | 2028 | 2.98 (2.45 - 3.62) | ARIMA |
| NH Asian or Pacific Islander | 2029 | 2.97 (2.41 - 3.67) | ARIMA |
| NH Asian or Pacific Islander | 2030 | 2.97 (2.38 - 3.70) | ARIMA |
| NH Asian or Pacific Islander | 2031 | 2.97 (2.37 - 3.72) | ARIMA |
| NH Asian or Pacific Islander | 2032 | 2.97 (2.35 - 3.74) | ARIMA |
| NH Asian or Pacific Islander | 2033 | 2.96 (2.35 - 3.75) | ARIMA |
| NH Asian or Pacific Islander | 2034 | 2.96 (2.34 - 3.75) | ARIMA |
| NH Asian or Pacific Islander | 2035 | 2.96 (2.33 - 3.76) | ARIMA |
| NH Black | 2026 | 7.64 (6.87 - 8.50) | ARIMA |
| NH Black | 2027 | 7.57 (6.60 - 8.67) | ARIMA |
| NH Black | 2028 | 7.51 (6.46 - 8.74) | ARIMA |
| NH Black | 2029 | 7.47 (6.36 - 8.76) | ARIMA |
| NH Black | 2030 | 7.43 (6.30 - 8.77) | ARIMA |
| NH Black | 2031 | 7.41 (6.26 - 8.76) | ARIMA |
| NH Black | 2032 | 7.38 (6.23 - 8.75) | ARIMA |
| NH Black | 2033 | 7.37 (6.21 - 8.74) | ARIMA |
| NH Black | 2034 | 7.36 (6.19 - 8.73) | ARIMA |
| NH Black | 2035 | 7.34 (6.18 - 8.73) | ARIMA |
| NH White | 2026 | 6.57 (6.11 - 7.06) | ARIMA |
| NH White | 2027 | 6.72 (5.93 - 7.62) | ARIMA |
| NH White | 2028 | 6.89 (5.80 - 8.18) | ARIMA |
| NH White | 2029 | 7.07 (5.73 - 8.72) | ARIMA |
| NH White | 2030 | 7.25 (5.68 - 9.26) | ARIMA |
| NH White | 2031 | 7.44 (5.66 - 9.80) | ARIMA |
| NH White | 2032 | 7.64 (5.65 - 10.33) | ARIMA |
| NH White | 2033 | 7.84 (5.65 - 10.88) | ARIMA |
| NH White | 2034 | 8.05 (5.67 - 11.43) | ARIMA |
| NH White | 2035 | 8.27 (5.69 - 12.00) | ARIMA |
| Northeast | 2026 | 4.01 (3.58 - 4.50) | ARIMA |
| Northeast | 2027 | 4.00 (3.45 - 4.62) | ARIMA |
| Northeast | 2028 | 3.98 (3.38 - 4.69) | ARIMA |
| Northeast | 2029 | 3.97 (3.34 - 4.72) | ARIMA |
| Northeast | 2030 | 3.96 (3.31 - 4.74) | ARIMA |
| Northeast | 2031 | 3.95 (3.29 - 4.75) | ARIMA |
| Northeast | 2032 | 3.95 (3.28 - 4.75) | ARIMA |
| Northeast | 2033 | 3.94 (3.27 - 4.76) | ARIMA |
| Northeast | 2034 | 3.94 (3.26 - 4.76) | ARIMA |
| Northeast | 2035 | 3.94 (3.26 - 4.76) | ARIMA |
| Midwest | 2026 | 6.51 (5.87 - 7.22) | ARIMA |
| Midwest | 2027 | 6.62 (5.53 - 7.92) | ARIMA |
| Midwest | 2028 | 6.67 (5.22 - 8.51) | ARIMA |
| Midwest | 2029 | 6.69 (4.96 - 9.02) | ARIMA |
| Midwest | 2030 | 6.70 (4.73 - 9.48) | ARIMA |
| Midwest | 2031 | 6.70 (4.53 - 9.90) | ARIMA |
| Midwest | 2032 | 6.70 (4.36 - 10.29) | ARIMA |
| Midwest | 2033 | 6.70 (4.21 - 10.67) | ARIMA |
| Midwest | 2034 | 6.70 (4.07 - 11.03) | ARIMA |
| Midwest | 2035 | 6.70 (3.95 - 11.37) | ARIMA |
| South | 2026 | 8.09 (7.46 - 8.76) | ARIMA |
| South | 2027 | 8.35 (7.45 - 9.35) | ARIMA |
| South | 2028 | 8.61 (7.50 - 9.90) | ARIMA |
| South | 2029 | 8.89 (7.57 - 10.44) | ARIMA |
| South | 2030 | 9.18 (7.67 - 10.98) | ARIMA |
| South | 2031 | 9.47 (7.78 - 11.52) | ARIMA |
| South | 2032 | 9.77 (7.91 - 12.08) | ARIMA |
| South | 2033 | 10.09 (8.04 - 12.66) | ARIMA |
| South | 2034 | 10.41 (8.19 - 13.24) | ARIMA |
| South | 2035 | 10.75 (8.34 - 13.85) | ARIMA |
| West | 2026 | 4.29 (3.86 - 4.78) | ARIMA |
| West | 2027 | 4.28 (3.72 - 4.92) | ARIMA |
| West | 2028 | 4.26 (3.64 - 5.00) | ARIMA |
| West | 2029 | 4.25 (3.59 - 5.04) | ARIMA |
| West | 2030 | 4.24 (3.55 - 5.07) | ARIMA |
| West | 2031 | 4.23 (3.52 - 5.09) | ARIMA |
| West | 2032 | 4.23 (3.51 - 5.10) | ARIMA |
| West | 2033 | 4.22 (3.49 - 5.10) | ARIMA |
| West | 2034 | 4.22 (3.48 - 5.11) | ARIMA |
| West | 2035 | 4.21 (3.48 - 5.11) | ARIMA |
| Urban | 2021 | 5.68 (5.34 - 6.04) | ARIMA |
| Urban | 2022 | 6.05 (5.34 - 6.86) | ARIMA |
| Urban | 2023 | 6.36 (5.25 - 7.71) | ARIMA |
| Urban | 2024 | 6.62 (5.10 - 8.58) | ARIMA |
| Urban | 2025 | 6.82 (4.92 - 9.44) | ARIMA |
| Urban | 2026 | 6.98 (4.73 - 10.30) | ARIMA |
| Urban | 2027 | 7.11 (4.52 - 11.16) | ARIMA |
| Urban | 2028 | 7.21 (4.33 - 12.00) | ARIMA |
| Urban | 2029 | 7.28 (4.13 - 12.83) | ARIMA |
| Urban | 2030 | 7.35 (3.95 - 13.65) | ARIMA |
| Urban | 2031 | 7.39 (3.78 - 14.46) | ARIMA |
| Urban | 2032 | 7.43 (3.62 - 15.26) | ARIMA |
| Urban | 2033 | 7.46 (3.47 - 16.06) | ARIMA |
| Urban | 2034 | 7.48 (3.32 - 16.84) | ARIMA |
| Urban | 2035 | 7.50 (3.19 - 17.62) | ARIMA |
| Rural | 2021 | 7.99 (7.25 - 8.80) | ARIMA |
| Rural | 2022 | 8.29 (7.23 - 9.50) | ARIMA |
| Rural | 2023 | 8.59 (7.27 - 10.16) | ARIMA |
| Rural | 2024 | 8.91 (7.34 - 10.81) | ARIMA |
| Rural | 2025 | 9.24 (7.44 - 11.47) | ARIMA |
| Rural | 2026 | 9.58 (7.56 - 12.15) | ARIMA |
| Rural | 2027 | 9.94 (7.69 - 12.84) | ARIMA |
| Rural | 2028 | 10.30 (7.84 - 13.55) | ARIMA |
| Rural | 2029 | 10.68 (7.99 - 14.28) | ARIMA |
| Rural | 2030 | 11.08 (8.16 - 15.05) | ARIMA |
| Rural | 2031 | 11.49 (8.33 - 15.84) | ARIMA |
| Rural | 2032 | 11.91 (8.52 - 16.66) | ARIMA |
| Rural | 2033 | 12.36 (8.72 - 17.52) | ARIMA |
| Rural | 2034 | 12.81 (8.92 - 18.40) | ARIMA |
| Rural | 2035 | 13.29 (9.13 - 19.33) | ARIMA |
| LCI = Lower Confidence Interval |  |  |  |
| UCI = Upper confidence Interval |  |  |  |
